# Ultrastructural pathological features and clinical significance of recurrent chordoma

**DOI:** 10.1016/j.jbo.2026.100805

**Published:** 2026-09-27

**Authors:** Qi Zhang, Hui Yang, Cuiping Zhang, Longtao He, Qing Chang

**Affiliations:** a Department of Ultrastructural Pathology, Neuropathology Center, Beijing Neurosurgical Institute, Beijing Tiantan Hospital, Capital Medical University, Beijing 100070, China; b Core Facilities Center, Capital Medical University, Beijing 100069, China; c Department of Neuropathology, Neuropathology Center, Beijing Neurosurgical Institute, Beijing Tiantan Hospital, Capital Medical University, Beijing 100070, China

**Keywords:** Recurrent chordoma, Transmission electron microscopy, Subcellular ultrastructure, Malignant progression, Prognostic stratification

## Abstract

Chordoma is a low-grade malignant osseous neoplasm characterized by an exceedingly high postoperative recurrence rate. Conventional histopathological assessment cannot identify early subcellular alterations that drive tumor recurrence, and the ultrastructural mechanisms mediating chordoma recurrence and malignant progression remain poorly delineated. This study was designed to characterise the distinct subcellular pathological features of recurrent chordoma and evaluate their clinical value in prognostic assessment and risk stratification. 50 surgically resected recurrent chordoma specimens were included as the recurrent cohort, with 50 contemporaneously collected primary chordoma specimens serving as the primary control cohort. Among these, 16 pairs consisted of matched TEM specimens from the same patient's primary and recurrent chordomas, while the remaining 34 pairs comprised non-matched primary and recurrent chordomas from different patients.; Transmission electron microscopy (TEM) was employed to systematically compare ultrastructural differences across cytoplasmic vacuolar patterns, organellar architecture, intercellular junctional complexes, nuclear morphology, and stromal microvascular ultrastructure, and the prevalence of each ultrastructural abnormality was analysed statistically. Compared with primary chordoma, recurrent lesions displayed extensive and progressive subcellular pathological remodelling, including atypical cytoplasmic vacuolation with giant cystic fusion, diffuse mitochondrial injury and rough endoplasmic reticulum degranulation, widespread disintegration of mitochondria-associated endoplasmic reticulum membrane (MAM) complexes, marked nuclear atypia, loss of intercellular desmosomal junctions, activation of invasive invadopodia, and stromal microvascular dysplasia. The prevalence of all these ultrastructural aberrations was significantly higher in the recurrent cohort than in the primary control cohort (all *P* < 0.001), and the severity of ultrastructural damage was positively correlated with the frequency of prior tumor recurrence. In this cross-sectional series, recurrent chordoma presents characteristic progressive ultrastructural lesions, in which organellar remodelling, loss of intercellular junctions, stromal microvascular abnormalities and nuclear atypia co-segregate with recurrent disease. These findings describe a subcellular morphological correlate of recurrence; because the present cohort was cross-sectional and did not correlate ultrastructural scores with survival, the value of TEM for recurrence-risk prediction, malignant grading or individualised treatment remains a hypothesis to be tested prospectively.

## Highlights

• Recurrent chordoma exhibits distinct progressive ultrastructural pathological remodelling compared with primary lesions.
• MAM disruption and invadopodia activation are core subcellular hallmarks of chordoma recurrence.
• The severity of ultrastructural impairment is positively correlated with the number of prior chordoma recurrence episodes.
• Ultrastructural examination provides adjunctive value for chordoma recurrence risk prediction and malignant stratification.

## 1 Background

Chordoma is a rare primary bone tumor originating from embryonic residual notochord tissue, predominantly involving the skull base, sacrococcygeal region and midline spine, and accounts for 1% to 4% of all primary bone tumours [1]. Although it grows indolently, chordoma is characterized by potent local invasiveness and a persistently high postoperative recurrence rate, which remains a major clinical challenge [2]. Clinical follow-up data indicate that the 5-year recurrence rate of patients undergoing surgical resection alone can reach 60% to 80%. Repeatedly recurrent chordomas frequently undergo dedifferentiation, malignant upgrade and even distant metastasis, representing the leading cause of poor long-term prognosis and shortened overall survival [3].

Currently, routine clinical diagnosis of chordoma relies mainly on light microscopic histopathology and immunohistochemical markers. Classic primary chordoma typically presents with vacuolated physaliferous cells and myxoid stroma under light microscopy, and the diagnostic system has been well established [4]. However, it is now recognised that a subset of apparently conventional, low-grade chordomas recur repeatedly after surgery, and a proportion undergo histological progression to a dedifferentiated or poorly differentiated phenotype that carries a markedly higher metastatic rate and shorter survival [1], [5], [6]. Conventional light microscopy only identifies morphological differences at the tissue level but cannot capture subtle pathological alterations at the subcellular level, making it difficult to clarify the morphological mechanisms underlying postoperative recurrence, invasion and progression of chordoma [7].

Transmission electron microscopy (TEM) allows direct visualisation of organelle configuration, intercellular junction patterns and functional subcellular complexes, and remains a useful ancillary tool in diagnostic surgical pathology when light microscopy and immunohistochemistry are inconclusive [8], [9]. Most prior ultrastructural studies of chordoma have examined primary tumours [10], [11], [12], and, to our knowledge, systematic TEM comparisons of primary versus recurrent lesions have not been reported. The characteristic subcellular pathological indicators specific to recurrent chordoma have not been defined, and the ultrastructural regulatory mechanisms underlying recurrence remain to be elucidated [9].

Against this background, this study systematically compared ultrastructural differences between primary and recurrent chordoma, summarized specific subcellular pathological features of recurrent chordoma, and explored the association between ultrastructural abnormalities and tumor recurrence, invasion and malignant transformation. The findings may reveal the core pathological mechanism of chordoma recurrence at the subcellular level, and provide morphological evidence for early prediction of recurrence risk, optimization of malignant grading criteria and screening of therapeutic targets.

## 2 Methods

### 2.1 Study subjects

A total of 100 surgically resected chordoma specimens were collected from the Department of Neuropathology of our hospital between January 2020 and May 2025, including 50 recurrent chordoma specimens (recurrent group) and 50 primary chordoma specimens (primary group). In the recurrent group, patient age ranged from 5 to 74 years (mean 45.1 ± 16.1 years), with 28 males and 22 females. Tumor locations included the clivus (*n* = 28), skull base (*n* = 7), sellar region (*n* = 8), cavernous sinus (*n* = 2), right parapharyngeal space (*n* = 1), and spine (*n* = 4). 37 cases had a single recurrence, and 13 cases had two or more recurrences. In the primary group, patient age ranged from 5 to 78 years (mean 38.7 ± 16.3 years), with 31 males and 19 females. Tumor locations included the clivus (*n* = 39), skull base (*n* = 6), and sellar region (*n* = 5).

All cases were definitively diagnosed by histopathology and immunohistochemistry. Cases complicated with other bone tumours, receiving preoperative radiotherapy or chemotherapy, with necrotic or hemorrhagic specimens, or with unqualified sampling were excluded. There were no statistically significant differences in age, gender or tumor location between the two groups (*P* > 0.05), indicating comparability.This retrospective study was approved by the Institutional Ethics Committee of Beijing Tiantan Hospital, Capital Medical University. The requirement for informed consent was waived, since only anonymized archival pathological specimens were used for ultrastructural observation, without access to patients' identifiable personal data. This study was performed in accordance with the principles of the Declaration of Helsinki.

Treatment history and the extent of resection were reviewed for all patients from operative reports, post-operative MRI and pathology margin reports. The 50 primary tumours were all first resections in patients who had received no neoadjuvant or adjuvant radiotherapy, chemotherapy, immunotherapy or other pre-operative treatment. In the recurrent cohort, every patient had at least one prior surgical resection. The extent of the most recent preceding operation was gross-total resection in 34 cases and subtotal/partial resection in 2 cases.; resection status could not be ascertained from transferred medical records for the remaining 14 cases. To exclude treatment-induced ultrastructural artefacts, the recurrent cohort was restricted to patients without intervening radiotherapy/chemotherapy/prior treatment status is listed in Table 1 and is acknowledged as a potential confounder. Subgroup analysis showed that the recurrent-group ultrastructural abnormalities were consistent across tumor locations and were more pronounced in patients with two or more recurrences than in those with a single recurrence; tumor location was not significantly associated with the severity of these changes (*P* > 0.05).

**Table 1:** Treatment history and resection characteristics of the study cohorts.

Characteristic	Primary tumor cohort (*n* = 50)	Recurrent tumor cohort (n = 50)
*Patient-matched paired specimens*		
Unpaired specimens	34 (68)	34 (68)
Paired specimens	16 (32)	16 (32)
*Prior surgical resection history*		
First resection (no prior surgery)	50 (100)	0 (0)
≥1 prior resection	0 (0)	50 (100)
*Number of prior recurrences*		
Single recurrence	0 (0)	37 (74)
≥2 recurrences	0 (0)	13 (26)
*Extent of most recent prior resection*		
Gross-total resection (GTR)	50 (100)	34 (68)
Subtotal / partial resection	0 (0)	2 (4)
Resection extent not documented in transferred medical records	0 (0)	14 (32)
*Intervening / prior treatment between resections*		
Radiotherapy (any)	0 (0)	2 (4)
Chemotherapy (any)	0 (0)	0 (0)
Immunotherapy (any)	0 (0)	0 (0)
Other preoperative / intervening treatment	0 (0)	0 (0)
No documentation intervening radiotherapy or chemotherapy/ Not definitively verifiable	0 (0)	48(96)
*Tumor location (subgroup analysis)*		
Distribution across locations (cortical / subcortical / deep / other)	As reported in text	As reported in text; no significant association with ultrastructural severity

### 2.2 Specimen preparation

All fresh surgical tumor specimens were sampled within 30 min after resection. Necrotic, hemorrhagic, fibrotic and inflammatory areas were avoided, and active proliferative tumor regions were selected and cut into 1 mm × 1 mm × 1 mm tissue blocks. Fresh tissue blocks were immediately fixed in 3% glutaraldehyde at 4 °C for 2 h, then rinsed with phosphate-buffered saline (PBS) 3 times for 10 min each. Post-fixation was performed with 1% osmium tetroxide for 1 h, followed by thorough rinsing with PBS. Graded ethanol and acetone were used for stepwise dehydration, and Epon 812 epoxy resin was applied for embedding and curing. Ultrathin sections of 70 nm thickness were prepared with an ultramicrotome, double-stained with uranyl acetate and lead citrate, and stored at room temperature in the dark for electron microscopic examination.

### 2.3 Transmission electron microscopy examination

All specimens were observed using a Hitachi H-7650 transmission electron microscope. Five clear high-power fields, excluding necrotic, haemorrhagic, fibrotic and inflammatory areas, were randomly selected per specimen. All fields were evaluated independently by two experienced neuropathologists who were blinded to group allocation; a given abnormality was scored as present when it was observed in at least three of the five fields. Inter-observer agreement was assessed with Cohen's kappa (kappa =0.79), and any disagreement was resolved by consensus with a third senior pathologist. The main observation indicators included overall tumor cell morphology, cytoplasmic vacuolar structure, morphology of mitochondria and rough endoplasmic reticulum, integrity of MAM complexes, nuclear morphology and chromatin distribution, intercellular desmosome junctions, cell membrane protrusions, and ultrastructure of stroma and microvessels.

Morphological differences of subcellular structures between the two groups were compared, and the incidence of each pathological ultrastructural abnormality was calculated.

### 2.4 Statistical analysis

Statistical analysis was performed using GraphPad Prism 8 software. Categorical data were expressed as n(%) and intergroup comparisons were performed using the chi-square test or Fisher's exact test for small samples. Normally distributed continuous data were expressed as mean ± standard deviation, and intergroup comparisons were performed using the independent samples *t*-test. A *P* value<0.05 was considered statistically significant.

## 3 Results

### 3.1 Ultrastructural features of primary chordoma

In the primary group, chordoma cells were regular in shape, mostly oval or polygonal, with loose and orderly arrangement. Scattered hemorrhagic foci and vascular structures were visible in the stroma (Fig. 1A). Tumor nuclei had regular morphology, smooth and continuous nuclear membrane, and evenly distributed chromatin (Fig. 1B). The cytoplasm contained abundant uniformly sized, well-defined droplet-like vacuoles (Fig. 1C), corresponding to the typical physaliferous cell morphology of chordoma. Mitochondria were intact with clear continuous cristae; rough endoplasmic reticulum was regularly arranged without dilation, vesiculation or ribosome detachment (Fig. 1D–E). MAM complexes were structurally intact and orderly distributed (Fig. 1F), indicating stable organelle function. Intercellular desmosome junctions were abundant and structurally complete (Fig. 1G). The cell membrane surface had slender regular microvilli without invasive invadopodia (Fig. 1H). The tumor stroma was rich in a homogeneous amorphous myxoid matrix, with intact regularly shaped blood vessels and no malformation or abnormal permeability (Fig. 1I).

**Fig. 1:**
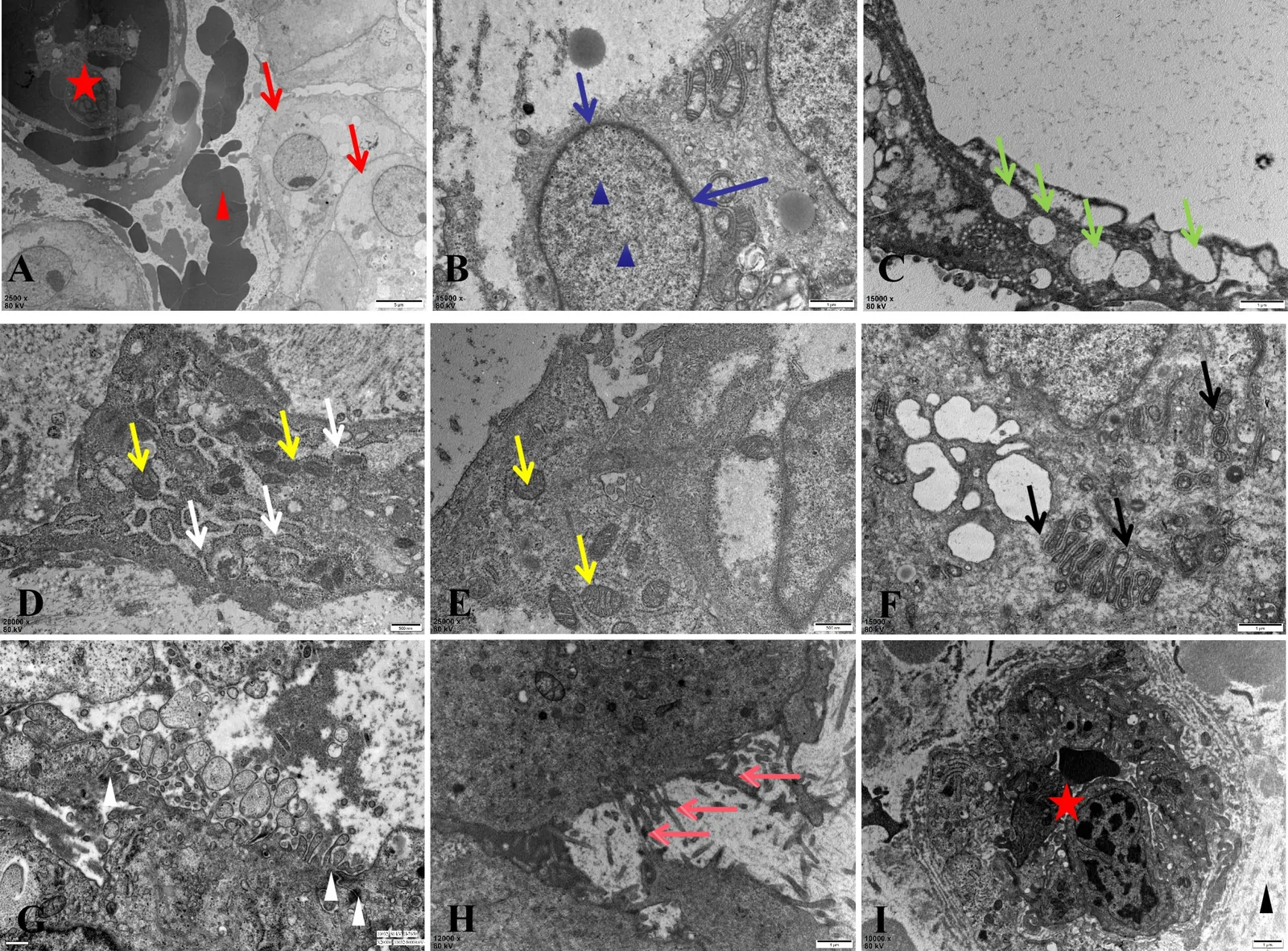
Ultrastructural features of primary chordoma. (A) Regular oval tumor cells with orderly arrangement. (Red arrows indicate tumor cells, red asterisk indicates blood vessels, and red triangle indicates hemorrhagic foci. Scale bar = 1 μm) (B) Regular nucleus with smooth nuclear membrane (Indicate by blue arrows) and evenly distributed chromatin (Indicate by blue triangle. Scale bar = 1 μm). (C) Uniform droplet-like vacuoles in the cytoplasm.(Indicate by green arrows. Scale bar = 1 μm) (D–E) Intact mitochondria with clear cristae and regularly arranged rough endoplasmic reticulum (RER). (Yellow arrows indicate mitochondria; white arrows indicate RER. Scale bar = 0.5 μm) (F) Intact mitochondria-associated endoplasmic reticulum membrane complexes. (Indicate by black arrows. Scale bar = 1 μm) (G) Abundant intact desmosome junctions between cells. (Indicate by white triangles. Scale bar = 0.5 μm) (H) Slender regular microvilli on cell membrane. (Indicate by pink arrows. Scale bar = 1 μm) (I) Homogeneous myxoid stroma (Indicate by black triangle. Scale bar = 1 μm) with intact vascular structure (Indicate by red asterisk. Scale bar = 1 μm). Note: The displayed image are from 7 independent primary chordoma patients. (For interpretation of the references to colour in this figure legend, the reader is referred to the web version of this article.)

### 3.2 Ultrastructural alterations in recurrent chordoma

34 unpaired and 16 paired recurrent chordoma cells exhibited extensive pathological remodelling and atypia, with significant differences from primary chordoma (Table 2, Table 3, Table 4). The detailed features are described below.

**Table 2:** Comparison of the incidence of ultrastructural abnormalities between the Unpaired Primary and Recurrent Chordoma [n (%)].

Ultrastructural abnormalities	Primary (*n* = 34)	Recurrent (n = 34)	χ2 value	*P* value
Atypical cytoplasmic vacuoles	8 (23.5)	32 (94.0)	34.971	<0.001
Mitochondrial swelling and cristae fracture	7 (20.6)	33 (97.1)	41.043	<0.001
Rough ER dilation/degranulation	4 (11.7)	32 (94.0)	46.278	<0.001
Disintegration of MAM complexes	3 (8.8)	33 (97.1)	53.125	<0.001
Nuclear atypia changes	9 (26.5)	33 (97.1)	35.868	<0.001
Reduction / loss of intercellular desmosome junctions	11 (32.3)	32 (94.0)	27.896	<0.001
Stromal and microvascular structural abnormalities	3 (8.8)	31 (91.2)	46.118	<0.001

**Table 3:** Paired comparative analysis of ultrastructural morphological features between the paired Primary and Recurrent Chordoma.

Ultrastructural morphological indicators	Primary (patient-matched specimens)	Recurrent (patient-matched specimens)
Cytoplasmic vacuolation	Mild and sparse vacuolation	Severe and extensive vacuolation
Mitochondrial morphology	Regular morphology and intact structure	Swollen, fractured and structurally disorganized
Rough endoplasmic reticulum morphology	Well-organized arrangement and intact lamellar structure	Dilated, disorganized and partially degranulated
Mitochondria-associated membrane (MAM) complex structure	Intact structure with tight connection	Structural disruption and loose connection
Intercellular junction integrity	Continuous and intact intercellular junctions	Fractured, incomplete and loosened junctions

**Table 4:** Comparison of the incidence of ultrastructural abnormalities between the paired Primary and Recurrent Chordoma [n (%)].

(A) Single recurrent of paired comparative analysis of ultrastructural morphological features				
Ultrastructural abnormalities	Primary (*n* = 7)	Single Recurrent (n = 7)	χ2 value	*P* value
Atypical cytoplasmic vacuoles	0 (0)	5 (71.4)	7.778	<0.05
Mitochondrial swelling and cristae fracture	1(14.3)	6 (86.0)	7.143	<0.05
Rough ER dilation/degranulation	2 (28.6)	6(86.0)	4.667	<0.05
Disintegration of MAM complexes	2 (28.6)	7 (100)	7.778	<0.05
Reduction/loss of desmosome junctions	2 (28.6)	5 (71.4)	2.571	Non-sense

(B) Recurrent chordoma with ≥2 recurrences of paired comparative analysis of ultrastructural morphological features				
Ultrastructural abnormalities	Primary (*n* = 9)	≥2 Recurrent (n = 9)	χ2 value	*P* value
Atypical cytoplasmic vacuoles	1 (11.1)	9 (100)	14.400	<0.001
Mitochondrial swelling and cristae fracture	1 (11.1)	9(100)	14.400	<0.001
Rough ER dilation/degranulation	2 (22.2)	9 (100)	11.4555	<0.001
Disintegration of MAM complexes	1 (11.1)	9 (100)	14.400	<0.001
Reduction/loss of desmosome junctions	3 (33.3)	9 (100)	9.000	<0.01

### 3.3 Cytoplasmic vacuole dysplasia

The overall vacuolization degree was markedly increased in the recurrent group, with highly variable vacuole size and irregular morphology (Fig. 2A–B). Numerous vacuoles fused to form giant cystic cavities with ill-defined boundaries (Fig. 2C). The internal matrix was disorderly degraded and sparsely vacuolated; the characteristic uniform droplet-like vacuolar structure seen in primary chordoma was completely destroyed (Fig. 2D). In some tumor cells, vacuoles completely disintegrated and the cytoplasm showed reticular loose degeneration (Fig. 2B).

**Fig. 2:**
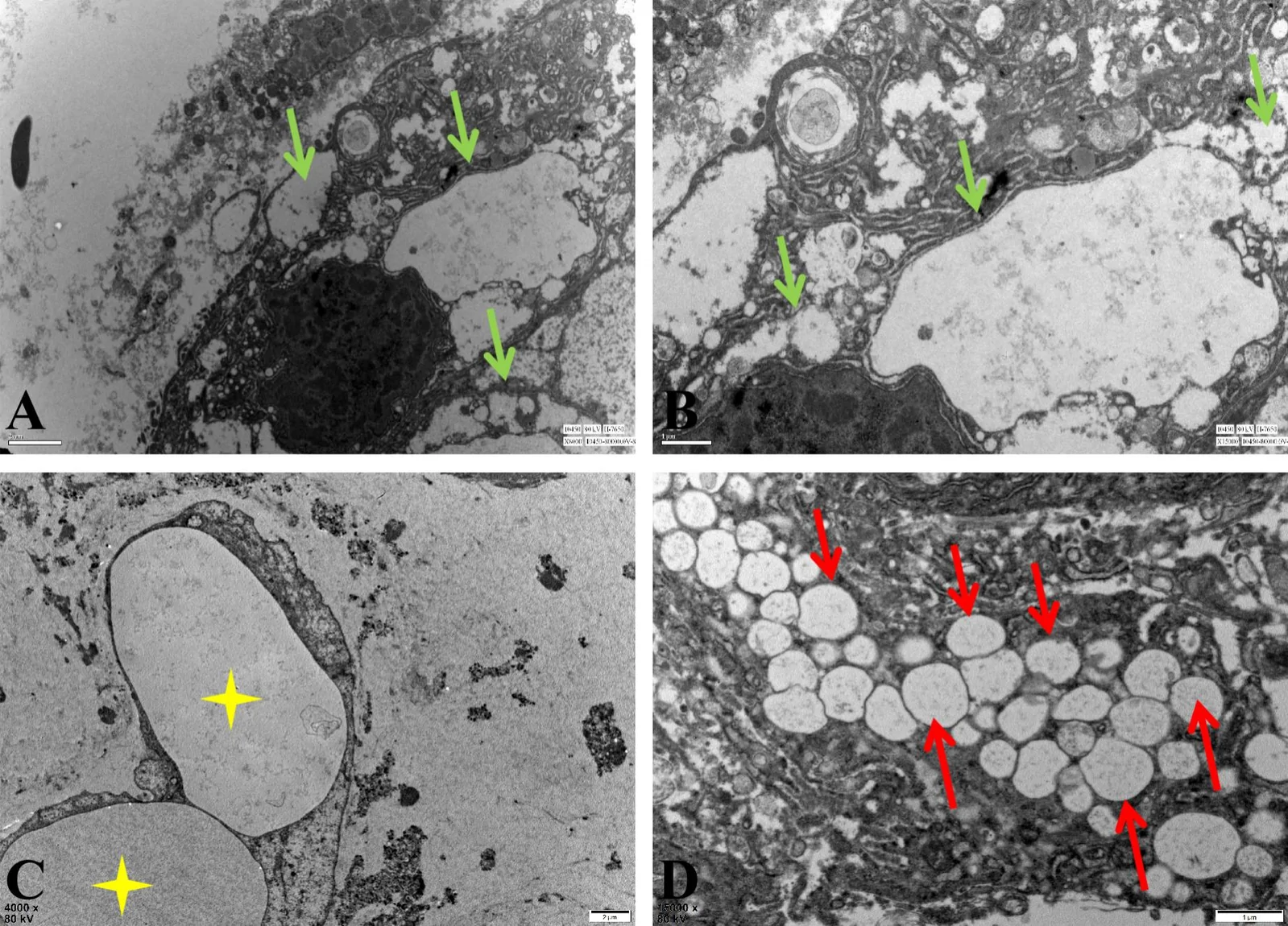
Pathological abnormalities of cytoplasmic vacuoles in recurrent chordoma. (A–B) Markedly uneven vacuole size and reticular loose degeneration of cytoplasm. (A, indicate by red arrows. Scale bar = 2 μm; B, indicate by green arrows. Scale bar = 1 μm). (C) Fusion of vacuoles into giant cystic cavities with blurred boundaries. (Indicate by yellow asterisks. Scale bar = 1 μm) (D) Comparison with uniform vacuoles in primary chordoma. (Indicate by red arrows. Scale bar = 1 μm) Note: The displayed image are from 3 independent recurrent chordoma patients. (For interpretation of the references to colour in this figure legend, the reader is referred to the web version of this article.)

The incidence of atypical cytoplasmic vacuoles was 96.0% (48/50) in the recurrent group, significantly higher than 16.0% (8/50) in the primary group (*P* < 0.001). Vacuole disintegration and cytoplasmic loose degeneration were more prominent in cases with two or more recurrences.

### 3.4 Organelle damage and MAM complex disintegration

Widespread organelle damage was observed in recurrent tumor cells. Mitochondria were generally swollen and deformed (Fig. 3A), with fractured and dissolved cristae (Fig. 3B), and some showed vacuolar degeneration (Fig. 3C). Rough endoplasmic reticulum was markedly dilated and vesiculated (Fig. 3D), with extensive ribosome detachment (Fig. 3E); the dilated cisternae contained abundant disordered flocculent deposits (Fig. 3F).

**Fig. 3:**
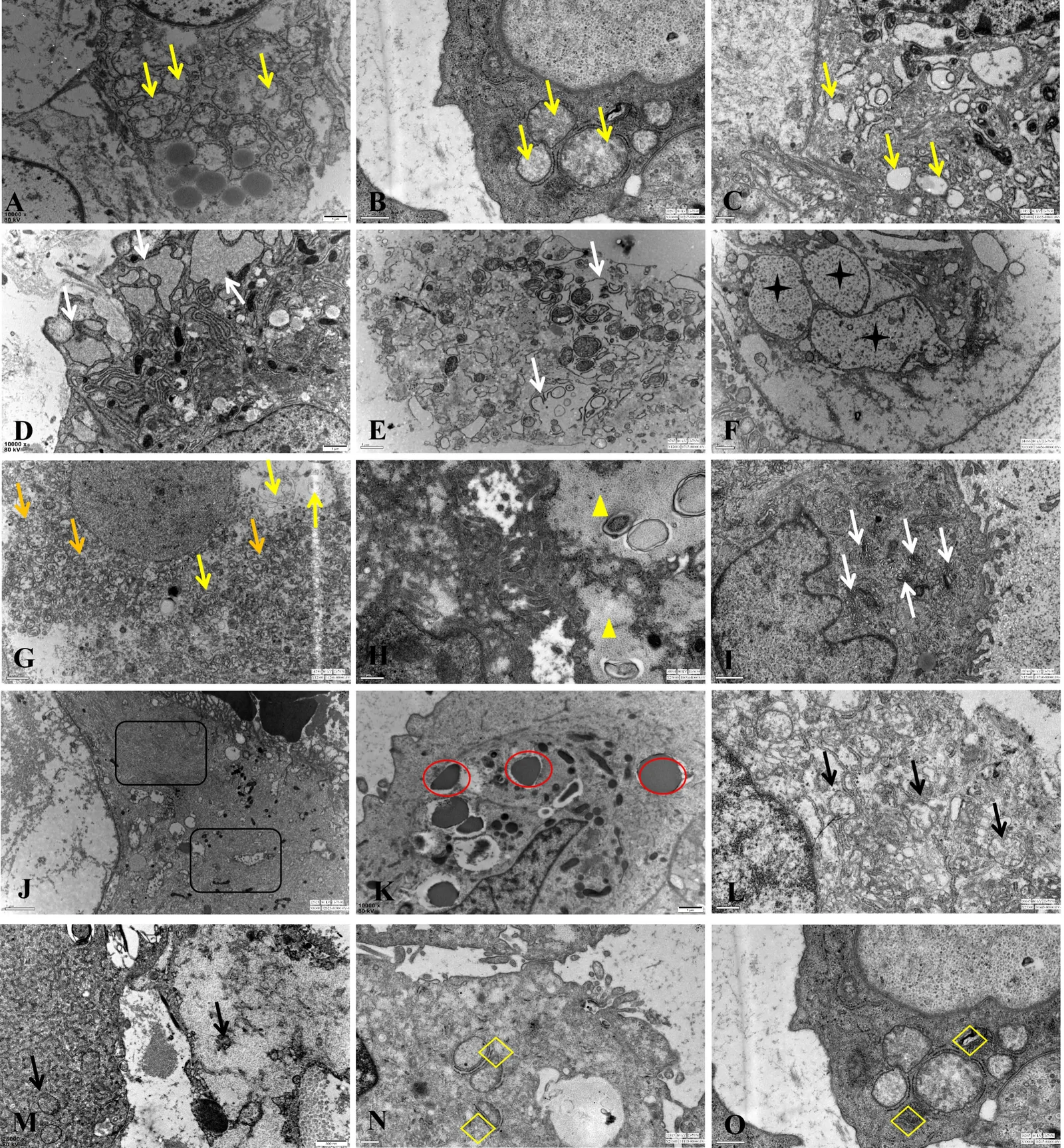
Organelle damage and disintegration of functional complexes in recurrent chordoma. (A) Swollen deformed mitochondria (indicated by yellow arrows. Scale bar = 1 μm) (B) Fractured and dissolved mitochondrial cristae. (Indicate by yellow arrows. Scale bar = 0.5 μm) (C) Vacuolar degeneration of mitochondria. (Indicate by yellow arrows. Scale bar = 0.5 μm) (D) Dilated and vesiculated rough endoplasmic reticulum. (Indicate by white arrows. Scale bar = 1 μm) (E) Ribosome detachment from rough endoplasmic reticulum. (Indicate by white arrows. Scale bar = 1 μm) (F) Flocculent deposits in dilated endoplasmic reticulum cisternae. (Indicate by black asterisk. Scale bar = 1 μm) (G) Mitophagosomes in cytoplasm. (Orange arrows indicate autophagosomes; yellow arrows indicate mitophagosomes. Scale bar =1 μm) (H) Glycogen lakes. (Indicate by yellow triangles. Scale bar =0.5 μm) (I) Increased Golgi apparatus.(Indicate by white arrows. Scale bar = 1 μm) (J) Abundant microfilaments. (Indicate by black squares. Scale bar = 2 μm) (K) Lipid droplet deposition. Indicate by red circles. Scale bar = 1 μm) (L–M) Disintegrated and disorderly arranged MAM complexes. (Indicate by black arrows. Scale bar = 0.5 μm) (N–O) Increased free ribosomes in cytoplasm.(Indicate by yellow squares. Scale bar = 0.5 μm) Note: The displayed image are from 12 independent recurrent chordoma patients. (For interpretation of the references to colour in this figure legend, the reader is referred to the web version of this article.)

Numerous autophagosomes, including mitophagosomes, were detected in the cytoplasm (Fig. 3G). Abundant glycogen aggregated to form glycogen lakes (Fig. 3H); the Golgi apparatus was increased in number (Fig. 3I), and massive microfilaments (Fig. 3J) and lipid droplets (Fig. 3K) were deposited.

The characteristic MAM complexes of primary chordoma were largely disintegrated, loosely structured and disorderly arranged in recurrent tumours (Fig. 3L–M). Intact complexes were observed in only 6% (3/50) of recurrent foci, significantly lower than 96% (48/50) in the primary group (*P* < 0.01). Meanwhile, free ribosomes in the cytoplasm were significantly increased (Fig. 3N–O), indicating abnormally elevated proliferative and metabolic activity [13].

### 3.5 Marked nuclear atypia

Tumor nuclei in the recurrent group were highly irregular (Fig. 4A). The nuclear membrane showed multiple deep folds, invaginations and notches, with interrupted continuity in some areas (Fig. 4B). Nucleoli were hypertrophic, increased in number and marginated toward the nuclear membrane (Fig. 4C). Chromatin was highly condensed, aggregated in clumps and unevenly distributed (Fig. 4D).

**Fig. 4:**
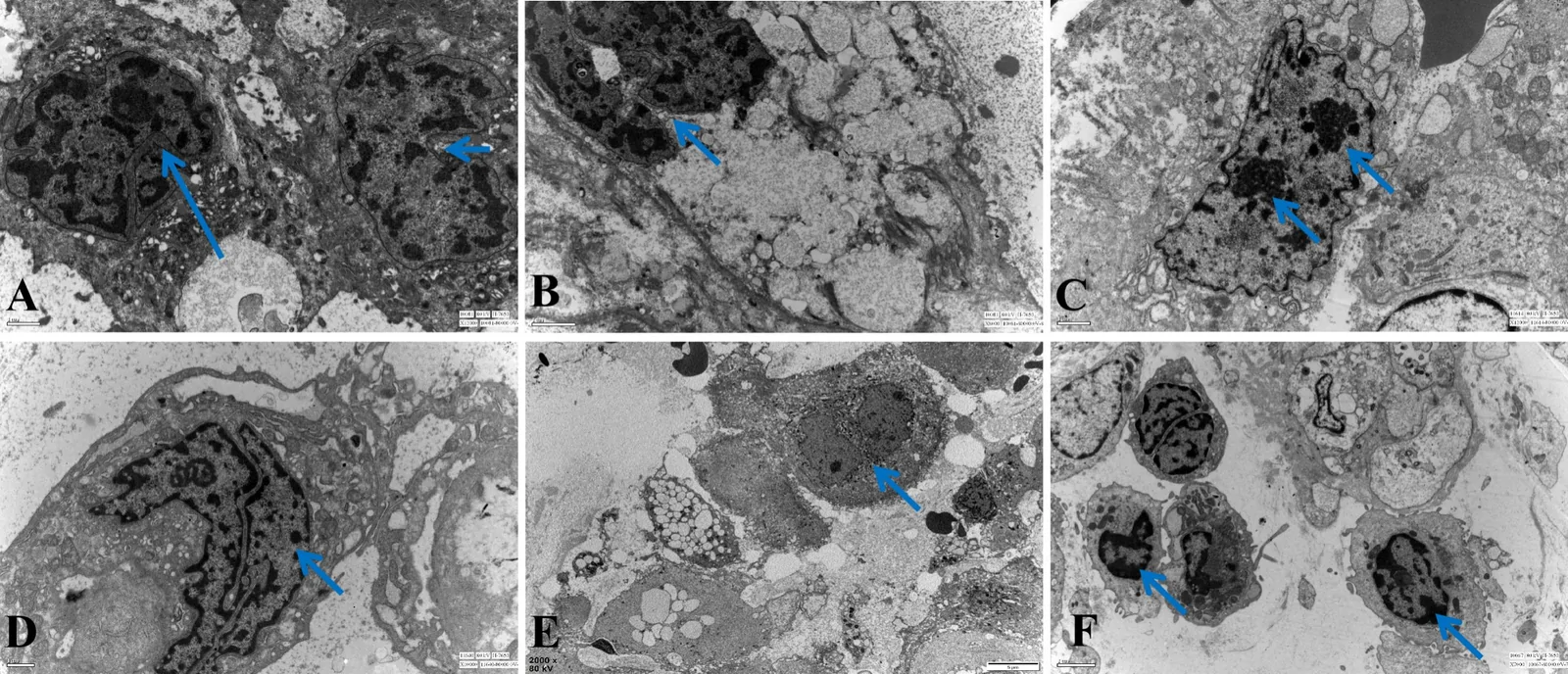
Nuclear atypia in recurrent chordoma. (A) Highly irregular nuclear morphology. (Indicate by blue arrows. Scale bar = 1 μm) (B) Deep folds and invaginations of nuclear membrane. (Indicate by blue arrows. Scale bar = 1 μm) (C) Hypertrophic marginated nucleoli. (Indicate by blue arrows. Scale bar = 1 μm) (D) Condensed clumped chromatin with uneven distribution.(Indicate by blue arrows. Scale bar = 1 μm) (E) Binucleated tumor cell. (Indicate by blue arrows. Scale bar = 5 μm) (F) Apoptotic cell. (Indicate by blue arrows. Scale bar = 2 μm) Note: The displayed image are from 4 independent recurrent chordoma patients. (For interpretation of the references to colour in this figure legend, the reader is referred to the web version of this article.)

Binucleated tumor cells (Fig. 4E) and apoptotic cells (Fig. 4F) were frequently observed, with marked variation in nuclear size and completely disorganized proliferation polarity. Nuclear atypia was positively correlated with recurrence times; the detection rate reached 100% (13/13) in cases with two or more recurrences, and the pathological damage was more severe than in single-recurrence cases [14], [15].

### 3.6 Degradation of intercellular junctions and activation of invasive structures

In the recurrent group, intercellular desmosome junctions were markedly reduced and structurally damaged, and completely absent in some regions (Fig. 5A), leading to increased tumor cell discreteness (Fig. 5B). The original slender microvilli on the cell membrane were largely shed (Fig. 5C), replaced by abundant blunt, irregular invasive invadopodia (Fig. 5D). The continuous activation of migration-and invasion-related structures indicated significantly enhanced tumor cell motility and invasiveness [16], [17].

**Fig. 5:**
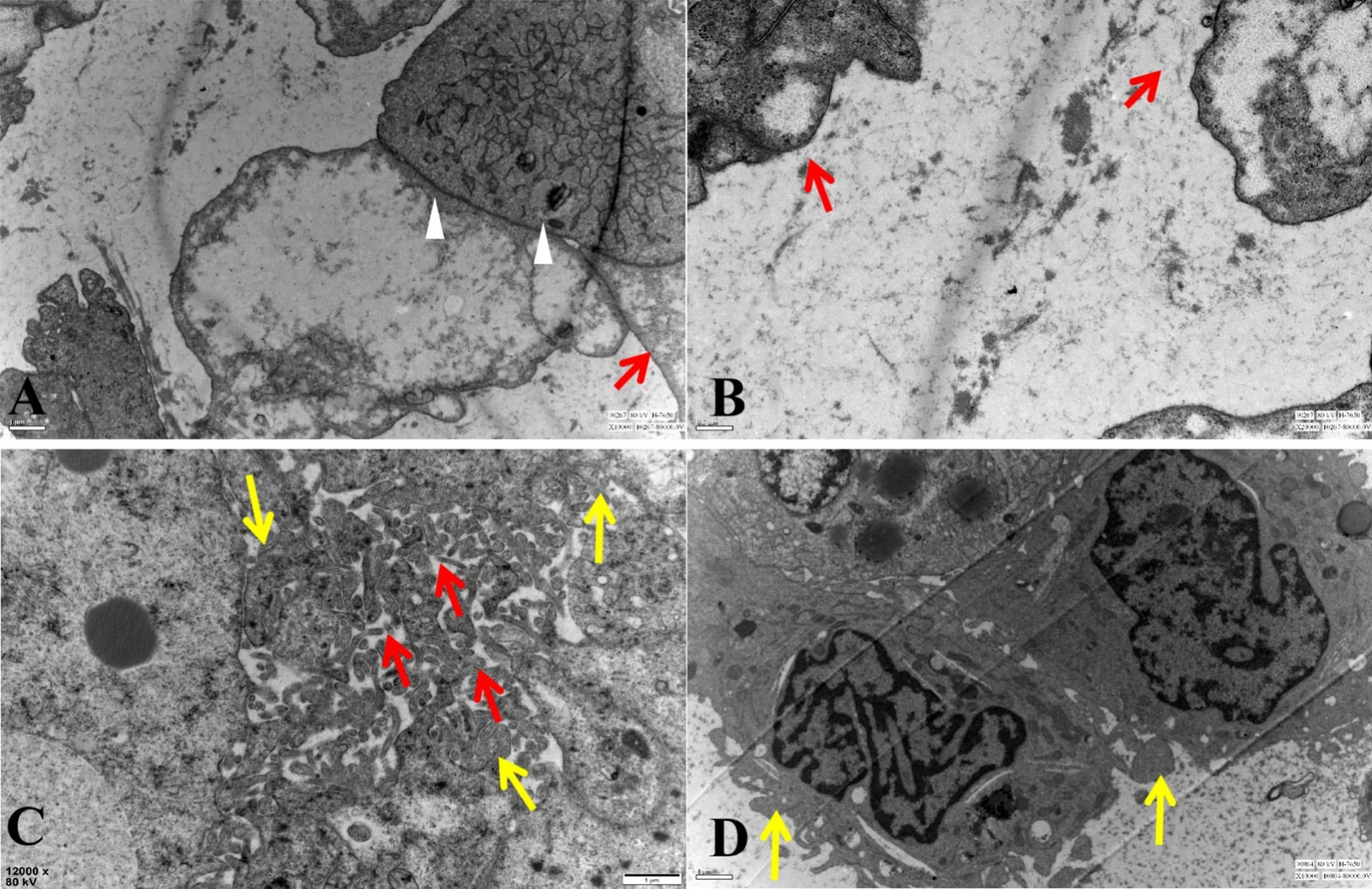
Degradation of intercellular junctions and activation of invasive structures in recurrent chordoma. (A) Reduced and damaged desmosome junctions. (Indicate by white triangles. Scale bar = 1 μm) (B) Increased discreteness of tumor cells. (Indicate by red arrows. Scale bar = 0.5 μm) (C) Shedding of surface microvilli. (Red arrows indicate abruption of microvilli; yellow arrows indicate blunt pseudopodia. Scale bar =1 μm) (D) Abundant invasive invadopodia on the cell membrane. (Indicate by yellow arrows. Scale bar =1 μm) Note: The displayed image are from 3 independent recurrent chordoma patients. (For interpretation of the references to colour in this figure legend, the reader is referred to the web version of this article.)

### 3.7 Stromal microstructure destruction and microvascular malformation

In the recurrent group, the myxoid stromal matrix was extensively degraded and lost (Fig. 6A), the overall stromal structure was loose and disorganized (Fig. 6B), and fiber bundles were disorderly arranged with fractured fibrosis and scattered collagen (Fig. 6C).

**Fig. 6:**
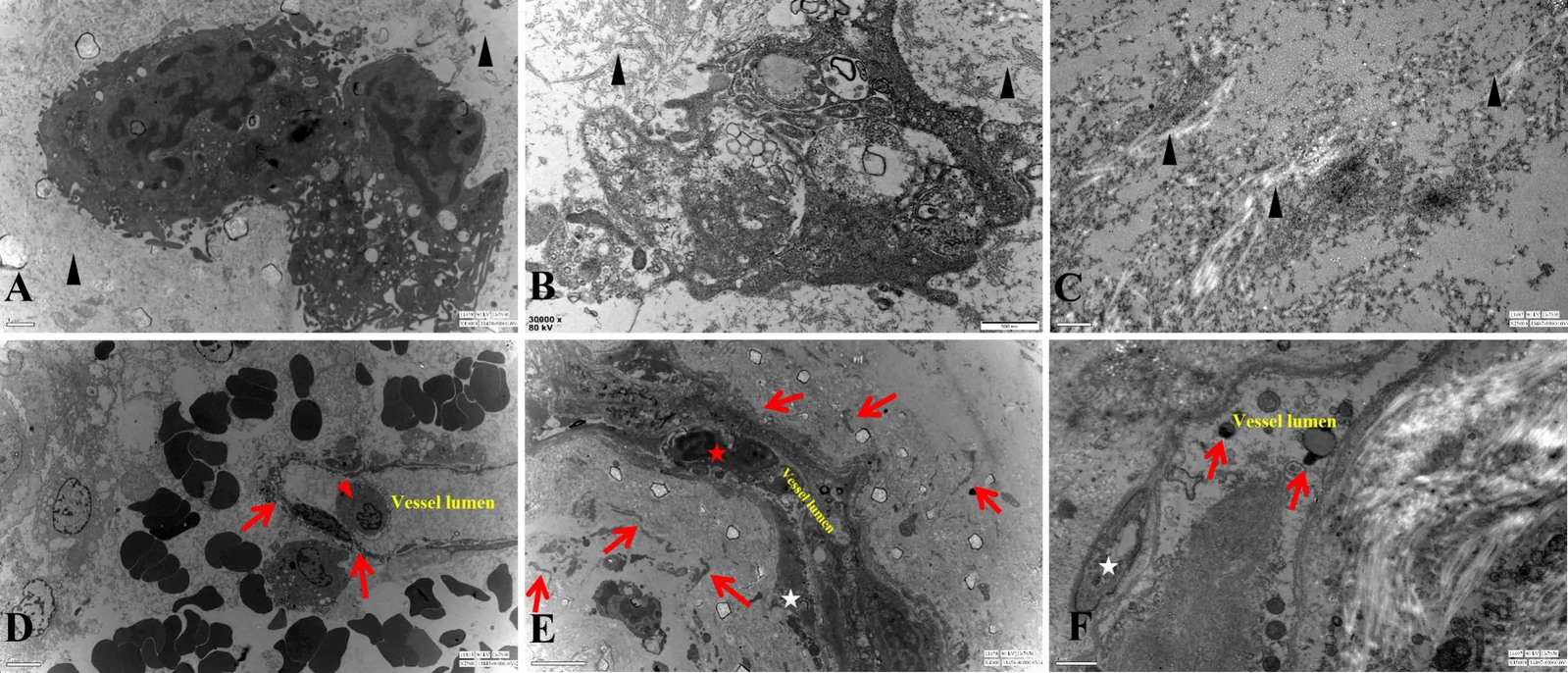
Stromal microstructure destruction and microvascular malformation in recurrent chordoma. (A) Degradation and loss of myxoid stromal matrix. (Indicate by black triangles. Scale bar = 1 μm) (B) Loose and disordered stromal structure. (Indicate by black triangles. Scale bar = 0.5 μm) (C) Disordered fractured fiber bundles. (Indicate by black triangles. Scale bar = 0.5 μm) (D) Thinned vascular wall with disrupted endothelial tight junctions. (Red arrows indicate thinned vascular wall, red triangle indicate lymphocyte. Scale bar = 5 μm) (E) Thinned vascular basement membrane with loose connections. (Red star indicates endothelial karyopyknosis and membranolysis; arrows; red arrow indicates vascular basement. Scale bar = 5 μm) (F) Chromatin loss in perivascular cells (Indicate by white star) and lysosomes in vascular lumen (Indicate by red arrows). Scale bar = 1 μm) Note: The displayed image are from 3 independent recurrent chordoma patients. (For interpretation of the references to colour in this figure legend, the reader is referred to the web version of this article.)

Stromal microvessels showed thinned walls, fractured or absent tight junctions between endothelial cells, and lymphocyte infiltration in the lumen (Fig. 6D). Endothelial cell nuclei were pyknotic, with disintegrated cell membranes. The vascular basement membrane was uniformly thinned, loosely connected to endothelial cells and pericytes, and extended outward away from the vessel wall (Fig. 6E). Some perivascular cells showed chromatin loss, and lysosomes were visible in the vascular lumen (Fig. 6F). Tumor vascular permeability was abnormally increased, with defective and fractured basement membrane in some microvessels, which constructed a favorable microenvironment for local infiltration and potential distant metastasis [18].

### 3.8 Comparison of ultrastructural abnormality incidence

Collectively, both paired and unpaired comparisons verified that recurrent chordoma tissues exhibited consistent and significant ultrastructural deterioration relative to their matched or unmatched primary tumours (Table 2, Table 3). These findings further illustrate that ultrastructural architectural damage is a prominent morphological characteristic of recurrent chordoma, which may be closely associated with tumor recurrence and malignant progression. Furthermore, a positive correlation was observed between the frequency of recurrence and the severity of subcellular organelle damage, indicating that ultrastructural destruction becomes more pronounced with multiple recurrences (Table 4A–B). The detection rates of all evaluated ultrastructural pathological lesions in the recurrent group were significantly higher than those in the primary group, with statistically significant differences observed across all indicators (all *P* < 0.05).

## 4 Discussion

### 4.1 Mechanisms of characteristic ultrastructural pathology in recurrent chordoma

This study confirmed via systematic transmission electron microscopy that recurrent chordoma exhibits specific progressive subcellular pathological damage distinct from primary chordoma. Persistent pathological remodelling of subcellular structures is the morphological substrate associated with repeated postoperative recurrence, local invasion and histological progression in this series [19].

Previous studies have demonstrated that regular vacuolar structure, intact MAM complexes and stable intercellular junctions are the subcellular morphological foundations for the indolent growth and low-grade malignancy of primary chordoma [10], [11], [12], [20]. Through comparative analysis, this study revealed that pathological ultrastructural changes in recurrent chordoma mainly involve four core dimensions: organelle dysfunction, loss of cell polarity, activation of invasive structures and imbalance of the tumor microenvironment. These co-occurring abnormalities are compatible with a coordinated subcellular programme associated with malignant behaviour in chordoma and other neoplasms [1], [2].

Cytoplasmic vacuolization is the most distinctive feature of chordoma, and in primary tumours it stably participates in cellular metabolism and osmotic homeostasis [10], [21]. In the recurrent tumours examined here, vacuoles were larger, more irregular and fused into giant cystic cavities, whereas the uniform droplet-like configuration seen in primary chordoma was lost. Whether this abnormality reflects altered metabolism or osmotic regulation cannot be resolved by TEM alone; the present observations are descriptive, and their mechanistic and prognostic significance remains to be determined [21], [22].

Mitochondria and the rough endoplasmic reticulum sustain energy supply, protein synthesis and the regulation of apoptosis, and MAM contacts between them coordinate Ca2+ signalling, lipid metabolism and cell-death decisions [23], [24], [25]. In the recurrent tumours we observed widespread mitochondrial swelling and cristae dissolution, RER degranulation and disintegration of MAM contacts. Because MAM remodelling has been implicated in metabolic adaptation, survival and proliferation in cancer [25], these ultrastructural changes are consistent with a dysregulated organellar state, although the present TEM data do not directly measure metabolism or apoptosis in chordoma.

Increased nuclear pleomorphism, prominent nucleoli and elevated mitotic activity have been associated with higher proliferative fractions and aggressive behaviour in chordoma [26]. At the molecular level, progression from conventional to recurrent or dedifferentiated chordoma is accompanied by the acquisition of TP53 alterations and other genomic changes and by epigenetic remodelling, which underlie loss of genomic stability and histological progression [5], [6], [27]. Our ultrastructural observations of marked nuclear atypia, particularly in multiply recurrent tumours, are concordant with these molecular findings. Loss of intercellular adhesive structures is a hallmark of tumor-cell dissemination: chordoma cells normally express E-cadherin/catenin complexes that maintain tissue cohesion [28], and the reduced desmosomal junctions and membrane protrusions observed here are ultrastructurally compatible with increased motility. Invadopodia-type protrusions are specialised cancer-cell structures that focally degrade extracellular matrix in vitro and in experimental models [16], [17]; their presence in recurrent chordoma is therefore consistent with an invasive phenotype, although TEM alone does not prove invasive behaviour in this series.

In addition, stromal matrix degradation and microvascular malformation not only supply sufficient nutrients for tumor proliferation, but also disrupt local tissue barriers and facilitate invasion and metastasis [22], [29]. This study further confirmed that more recurrences correlate with more severe subcellular malignant damage, supporting a vicious cycle of “surgical recurrence-subcellular malignant transformation–enhanced invasiveness – further recurrence” in chordoma [30].

### 4.2 Clinical value of ultrastructural pathology

The current diagnostic system for chordoma has notable limitations. Conventional histopathology only enables macroscopic subtype classification and cannot identify early recurrence potential or subclinical malignant transformation. Immunohistochemistry only reflects single protein expression and cannot comprehensively evaluate invasive activity and recurrence risk from a morphological perspective. Consequently, some apparently low-grade primary chordomas relapse unexpectedly after surgery, and the accuracy of current prognostic prediction remains limited [26], [27].

The characteristic ultrastructural indicators of recurrent chordoma identified in this study can compensate for the shortcomings of conventional pathological detection and have considerable clinical translational value.

Whether the ultrastructural features described here can stratify recurrence risk cannot be concluded from the present cross-sectional cohort, because tumours with these features were not shown to recur more often. We therefore advance this as a testable hypothesis: if a prospective cohort confirms that early subcellular lesions (for example, incipient MAM disruption, vacuolar atypia and junctional degeneration) precede recurrence, patients showing them might be considered for intensified follow-up. This remains speculative and requires prospective validation before it can guide management [7], [31].

The light-microscopic classification of chordoma distinguishes conventional, chondroid, dedifferentiated and poorly differentiated subtypes [1], [4] but does not provide real-time information on subcellular activity. We observed that the severity of ultrastructural abnormalities increased with the number of previous recurrences, which makes these features candidate descriptive markers of progressive histological change. We do not yet propose them as a new subcellular grading system, and treatment decisions-extent of resection, adjuvant radiotherapy and, where indicated, systemic therapy-should continue to follow current guidelines and multidisciplinary assessment [3], [32].

For therapeutic target development, core pathological features including MAM deconstruction, cell junction loss and stromal microvascular abnormalities reveal key structural targets for malignant progression. Targeted strategies focusing on subcellular structure repair, cell adhesion reconstruction and tumor microenvironment homeostasis may effectively inhibit proliferation and invasion and break the recurrence cycle, providing morphological theoretical support for new drug development and targeted therapy of refractory recurrent chordoma [23], [24].

Because the present study did not correlate ultrastructural scores with recurrence-free or overall survival, we cannot claim that the features described here independently predict outcome; they are reported here as descriptive markers of recurrent disease. At the population level, however, dedifferentiated and poorly differentiated chordoma - histological counterparts of marked progression-do carry higher metastatic risk and shorter survival than conventional chordoma [1], [33].

### 4.3 Study limitations and perspectives

Several limitations of this work should be acknowledged. Matched primary–recurrent pairs were scarce in our cohort, and a substantial proportion of tumours had no corresponding counterpart; to preserve statistical power we therefore supplemented the paired design with unpaired comparisons between the primary and recurrent cohorts. Because such cross-sectional contrasts cannot fully account for inter-individual heterogeneity and baseline clinicopathological differences (tumor site, interval between operations, prior radiotherapy), this hybrid approach may have introduced residual confounding. Transmission electron microscopy interrogates only a minute fraction of tumor volume, semi-quantitative grading of subcellular injury is observer-dependent, and the retrospective single-centre design without an independent validation set restricts generalisability [9]. Moreover, the study was descriptive and was not powered to determine whether the documented ultrastructural derangements precede aggressive behaviour or instead reflect its consequences.

Accordingly, we recommend prospective validation in future studies to determine whether these ultrastructural parameters are associated with adverse clinical outcomes, including shorter progression-free and overall survival, earlier local recurrence, and resistance to adjuvant therapy. Multi-centre cohorts with standardised tissue processing, blinded replicate scoring with assessment of inter-observer agreement, and time-to-event modelling adjusted for established clinicopathological covariates would allow derivation and external validation of a dedicated ultrastructural risk score for recurrent chordoma [34]. In parallel, integrating correlative light–electron microscopy with genomic and proteomic profiling should clarify the regulatory circuits that generate these subcellular abnormalities. Together, these steps would convert qualitative ultrastructural pathology into a reproducible, mechanism-anchored framework for risk stratification and targeted drug development in chordoma [35].

## 5 Conclusions

Chordoma recurrence is accompanied by a stepwise reorganisation of subcellular architecture that has no counterpart in the matched primary lesion [36]. In recurrent specimens we found atypical cytoplasmic vacuolation, diffuse organelle injury, fragmentation of MAM complexes, nuclear atypia, loss of intercellular junctional complexes and malformed stromal microvessels. Images were assigned according to a pre-specified sampling rule: five high-power fields free of necrosis and haemorrhage were selected at random in each case, and a feature was recorded as present only when it appeared in three or more fields. This scheme yields presence/absence frequencies rather than graded measurements. The abnormalities listed above should therefore be read as co-occurring morphological traits whose combined effect may facilitate postoperative relapse, local infiltration and malignant progression [25], [37]; no dose-response relation between lesion severity and outcome can be inferred from the prevalence data reported here. Within these limits, transmission electron microscopy captures early subcellular derangements linked to recurrence that are usually missed by routine histology and immunohistochemistry, and it may therefore serve as an adjunct for risk stratification, grading refinement, treatment tailoring and prognostication in recurrent chordoma [38], [39]. We acknowledge semi-quantitative scoring as a constraint; derivation of a continuous (0–3) severity score for each feature, formal assessment of inter-observer reproducibility and validation against progression-free and overall survival are stated as explicit aims in the Limitations section.

## Authorship confirmation

All authors listed on the manuscript meet the International Committee of Medical Journal Editors (ICMJE) criteria for authorship. Each author has made substantial contributions to the conception and design of the work, the acquisition, analysis and interpretation of data, and the drafting and critical revision of the manuscript for important intellectual content. All authors have approved the final version of the manuscript and agree to be accountable for all aspects of the work.

## Previous presentation

This manuscript has not been published previously and is not under consideration for publication elsewhere, in whole or in part.

## CRediT authorship contribution statement

**Qi Zhang:** Writing – review & editing, Writing – original draft, Methodology, Investigation, Funding acquisition, Formal analysis, Data curation, Conceptualization. **Hui Yang:** Validation, Investigation, Formal analysis, Data curation. **Cuiping Zhang:** Validation, Investigation, Data curation. **Longtao He:** Resources, Investigation. **Qing Chang:** Writing – review & editing, Resources, Project administration, Funding acquisition, Conceptualization.

## Informed consent

The requirement for written informed consent was waived by the Institutional Ethics Committee, as the study used only anonymised archival pathological specimens with no access to patients' identifiable personal data, and posed no additional risk to patients.

## Consent for publication

Not applicable. No identifiable individual patient data or images are included in this manuscript.

## Ethics approval

This retrospective study was reviewed and approved by the Institutional Ethics Committee of Beijing Tiantan Hospital, Capital Medical University. All procedures performed in the study were in accordance with the ethical standards of the institutional research committee and with the 1964 Declaration of Helsinki and its later amendments.

## Funding statement

This work was supported by the China University Industry-Research-Innovation Fund — Innovative Project for Next-Generation Information Technology (Grant No. 2024IT020). The funding body had no role in study design, data collection and analysis, decision to publish, or preparation of the manuscript.

## Funding

This work was supported by China University Industry-Research-Innovation Fund — Innovative Project for Next-Generation Information Technology (2024IT020).

## Declaration of competing interest

The authors declare that they have no known competing financial interests or personal relationships that could have appeared to influence the work reported in this paper.

## Data Availability

The datasets generated and/or analysed during the current study are available from the corresponding authors (Qing Chang, changqing055@bjni.org.cn; Qi Zhang, zhangqi5706@163.com) on reasonable request.
